# Attitudes Toward Signing Avatars Vary Depending on Hearing Status, Age of Signed Language Acquisition, and Avatar Type

**DOI:** 10.3389/fpsyg.2022.730917

**Published:** 2022-02-10

**Authors:** Lorna C. Quandt, Athena Willis, Melody Schwenk, Kaitlyn Weeks, Ruthie Ferster

**Affiliations:** Educational Neuroscience, Gallaudet University, Washington, DC, United States

**Keywords:** sign language, avatars, signing avatars, deaf, virtual humans

## Abstract

The use of virtual humans (i.e., avatars) holds the potential for interactive, automated interaction in domains such as remote communication, customer service, or public announcements. For signed language users, signing avatars could potentially provide accessible content by sharing information in the signer's preferred or native language. As the development of signing avatars has gained traction in recent years, researchers have come up with many different methods of creating signing avatars. The resulting avatars vary widely in their appearance, the naturalness of their movements, and facial expressions—all of which may potentially impact users' acceptance of the avatars. We designed a study to test the effects of these intrinsic properties of different signing avatars while also examining the extent to which people's own language experiences change their responses to signing avatars. We created video stimuli showing individual signs produced by (1) a live human signer (Human), (2) an avatar made using computer-synthesized animation (CS Avatar), and (3) an avatar made using high-fidelity motion capture (Mocap avatar). We surveyed 191 American Sign Language users, including Deaf (*N* = 83), Hard-of-Hearing (*N* = 34), and Hearing (*N* = 67) groups. Participants rated the three signers on multiple dimensions, which were then combined to form ratings of Attitudes, Impressions, Comprehension, and Naturalness. Analyses demonstrated that the Mocap avatar was rated significantly more positively than the CS avatar on all primary variables. Correlations revealed that signers who acquire sign language later in life are more accepting of and likely to have positive impressions of signing avatars. Finally, those who learned ASL earlier were more likely to give lower, more negative ratings to the CS avatar, but we did not see this association for the Mocap avatar or the Human signer. Together, these findings suggest that movement quality and appearance significantly impact users' ratings of signing avatars and show that signed language users with earlier age of ASL acquisition are the most sensitive to movement quality issues seen in computer-generated avatars. We suggest that future efforts to develop signing avatars consider retaining the fluid movement qualities integral to signed languages.

## Introduction

Virtual human avatars who use signed languages could improve digital infrastructure for accessing information, learning signed languages, or other aspects of signed interactions (Naert et al., 2020), especially in situations when face-to-face communication is not possible. Signing avatars could also help disseminate emergency-related information quickly and uniformly throughout a community in the case of evacuations, public health crises, or missing person alerts. While signing avatars are unlikely ever to match the responsiveness and natural movements of an actual human signer, signing avatars have potential benefits for increasing accessibility and use of signed languages in everyday life. For instance, the flexibility and interactive nature of signing avatars mean that an avatar's signing speed or appearance can be changed, content can be repeated on demand, and simple interactions can be programmed according to user needs. With sufficient development, signing avatars can allow semi-automated interaction, much like the ubiquitous customer service chat-bots which have become common in recent years. In this paper, we describe the results of a sizeable online rating study to examine the determinants of American Sign Language (ASL) users' responses to different types of signing avatars.

With appropriate scaling and development of digital tools, signing avatars could be used across various domains when live signers are unavailable or impossible to use. For instance, signing avatars could be used to translate content on a website automatically (Kennaway et al., 2007), translate educational content alongside a textbook (Adamo-Villani and Anasingaraju, 2016), provide time-sensitive information in public spaces (e.g., flight updates at an airport), or teach signed language lessons in virtual reality (Quandt et al., 2020). In these situations, the benefits of signing avatars over pre-recorded actual human signed videos include the ease of editing and automatic production of new signed information. However, sign language users are likely to have different opinions, or acceptance, of signing avatars in some spaces compared to others, and not all potential uses or engineering approaches of signing avatars will be worth the investment of research, time, and money. Additionally, some uses of signing avatars may be geared toward non-signers, such as using avatars for signed language instruction in virtual or mixed reality (Quandt et al., 2020; Shao et al., 2020), and developers creating avatars for different populations should be mindful of how different groups respond to different avatars. It is critical to note that the use of signing avatars may not be feasible, appropriate, or worthwhile in many situations (e.g., formal education, providing interpreting in face-to-face meetings; WFD and WASLI, 2018).

However, it is essential to identify the specific situations in which signing avatars can benefit and provide added value. Gaining a firm understanding of what makes a signing avatar comprehensible and likable will help guide future development in the field. Preliminary work has shown that deaf signers would welcome signing avatars in public locations where information-sharing is vital (e.g., train stations, hospitals; Kennaway et al., 2007). One area that could benefit from signing avatars is some health settings where patients share confidential information with a provider, such as during psychological assessments. Interaction with signing avatars in situations where a person may not want to divulge information to multiple parties would allow a signer to minimize the number of people involved in their care, which may ease discomfort in the presence of interpreters (Barber et al., 2010).

Developers can create signing avatars using several different processing pipelines. One of the significant distinctions between types of virtual human animation depends on whether the movement is based on recorded motion capture from actual human signers or whether the movements are based on computer-synthesized motions, programmed to result in signed language production. In the latter case of computer-synthesized motions, developers can use manual or automatic keyframe animation. Each of these engineering approaches has significant benefits and drawbacks (Gibet, 2018; Naert et al., 2020), and each approach has seen significant progress in recent years. Motion capture recordings tend to provide more realistic human movements (Alexanderson and Beskow, 2015; Quandt et al., 2020) but require costly investments of time to process and clean. Computer synthesized animations are more efficient since they can be programmed but result in a limited and less natural movement of the hands and reduced fluency in meaningful facial expressions. More recent innovations use machine learning to generate highly realistic signs from estimations of skeletal poses trained on existing video data (Saunders et al., 2020; Stoll et al., 2020). These newer animations are pushing the envelope of how accurate and realistic synthesized signing can look, and as such, it is more important than ever to assess how different factors affect end-users' views of signing avatars.

As signing avatars have continued to gain traction in recent years as a potentially powerful accessibility tool for signed language users, researchers have examined which intrinsic or extrinsic factors may contribute to how users perceive and accept the avatars. Intrinsic factors include characteristics of the avatar itself: appearance, type of movement, or facial expressions. Extrinsic factors that may impact receptivity or comprehension include the user's own fluency with a signed language, their attitudes toward technology, or their language and education history. Prior research has examined many of these factors (Kacorri et al., 2015) and identified specific factors which appear to be most important for predicting the reaction to signing avatars. Several prior studies have examined how signers perceive and rate signing avatars, using self-reported ratings and eye-tracking metrics (Huenerfauth and Kacorri, 2016). Prior work has shown that grammaticality ratings and natural motion can be associated with specific areas of eye fixation (Huenerfauth and Kacorri, 2016).

General attitudes toward signing avatars are best assessed in the context of other viable alternatives. For instance, one could compare signing avatars to written text or a video showing a human signer to examine how people react to different information-sharing modes in a specific context. One research study compared human and avatar signers who presented math problems to Deaf young adults and compared their performance and attitudes toward the signers (Hansen et al., 2018). While participants all preferred the human signer, their mathematics performance was equal in response to questions posed by both signers. Users reported dissatisfaction with the avatar's lack of facial and body expression, and across many research studies, overall appearance and facial expressions are critical considerations for acceptable signing avatars.

Intrinsic characteristics of signing avatars themselves can influence how potential users respond to them. These aspects could include appearance, form, movement, or details regarding their production of signed language. Virtual human characters can range in appearance from highly realistic to highly stylized or cartoonish, and these appearances may impact users' reactions to the avatars. While appearance alone could impact users' comprehension of signing avatars, one study found that was not the case, and legibility was the same between highly realistic and stylized signing avatars (Adamo-Villani et al., 2015). However, comprehension is not the only important measurement for a signing avatar. People's emotional, holistic impressions of the characters may also be critical for successful interaction with a signing avatar, going beyond simply the need to comprehend signs. In the Adamo-Villani et al. study, users found the stylized avatar significantly more appealing than the realistic avatar, likely reflecting the effect of the uncanny valley (Mori et al., 2012; Shin et al., 2019). In another study, users preferred natural-style avatars over anime-style avatars (Brock et al., 2018). These subjective measures of preference and attitude toward avatars provide valuable insight into the multidimensional factors that impact avatars' success.

Extrinsic factors, such as the viewer's own language use and hearing status, may also contribute to the acceptance of and responses to signing avatars. Given the wide diversity of people who use American Sign Language, individual differences may profoundly change how people view and respond to signing avatars. For instance, prior work suggests that the language environment in which a sign language user grows up, or their level of fluency with ASL, may impact their likelihood of responding positively or negatively to signing avatars—for instance, more use and knowledge of ASL predicts harsher ratings of signing animations (Kacorri et al., 2015). The type of school that the signer attended also was correlated with subjective judgments of signing animation—attending a residential Deaf school (where everyone's primary mode of communication is signed language) was linked to harsher ratings of signing animations. Thus, both intrinsic and extrinsic factors are critical when developing and deploying signing avatars in any sector of society.

While other work has focused on either the intrinsic factors or extrinsic factors driving attitudes toward signing animations, here we attempted to identify both kinds of factors. For instance, if higher ASL proficiency is related to harsher criticisms of a signing avatar—is that true for different kinds of signing avatars?

Our research group has developed a motion-capture-based signing avatar for use in an immersive virtual reality ASL learning system. During development, we identified key features of the signing avatar that would teach users basic ASL signs (Quandt et al., 2020). Through prior work on signing avatars and embodied learning, we designed an ASL teacher avatar that has the following features (1) produce fluid, biologically plausible movements that resemble native ASL signers' movements as much as possible; (2) display the facial expressions critical to correct ASL grammar, in a manner as close as possible to a native ASL signer; (3) be aesthetically pleasing, falling at the right point on the cartoon-to-realistic spectrum of animation styles; and (4) present as an “ideal” ASL teacher—knowledgeable, competent, kind, and professional. Using these guiding principles, we sought to examine intrinsic and extrinsic factors contributing to users' reactions to signing avatars in the current study. For signing avatars to gain traction and acceptance in online or community spaces (e.g., signing avatars providing information in a train station), it will be critical to understand who will be most likely to attend to signing avatars, as well as identifying what features of the signing avatars will make them most successful.

Our pre-registered hypothesis was that signing avatars created from motion capture recordings would elicit more positive attitudes and impressions, higher comprehension, and more natural signing ratings than computer-synthesized avatars (https://aspredicted.org/3e5hg.pdf). We also examined how ratings of the avatars varied based on hearing status, age of sign language acquisition, and self-reported ASL fluency measures. Analyzing the data through these multiple types of analyses allows us to better understand the effects of hearing status, language environment, culture, and fluency—as they relate to signers' perceptions of signing avatars. Past work has suggested that both a signer's fluency and their language environs, such as whether they attended a residential school for Deaf children, affect how signers rate avatars (Kacorri et al., 2015). However, prior work has not gathered large datasets from a wide variety of signers rating different types of signing avatars. Given the tremendous amount of variability in ASL users' language backgrounds and cultural identities and the proliferation of different types of signing avatars, we designed a study to capture more of this variability. Based on prior work, we predicted that a younger age of acquisition and higher ASL fluency would result in less favorable views of signing avatars overall, whereas people who learn ASL later in life or are less fluent will be more accepting of, and give higher ratings to, motion-capture avatars.

To test these hypotheses, we conducted an online rating survey in which 184 ASL users rated two different types of signing avatars on an array of different dimensions, along with real human video control. We chose these three signers to sample the many possible signing avatars developed to date. We especially wanted to compare two different processing pipelines against a human benchmark, so we included one motion capture avatar (Mocap) and one computer-synthesized avatar (CS). We recruited a sample of raters from across the country, with a wide range of variations in ASL fluency, including deaf, hard-of-hearing, and hearing ASL users.

## Methods

### ASL Signs

We selected eight individual ASL signs as the stimuli for this experiment: MILK, FRENCH-FRIES, TOILET, LIBRARY, SPAGHETTI, BACON, MUSEUM, and WEDDING (links provide corpus representations of the signs, Caselli et al., 2016). The first four signs listed are produced with one hand, whereas the last four are symmetrical signs produced using two hands.

### Video Stimuli

**Figure 1:**
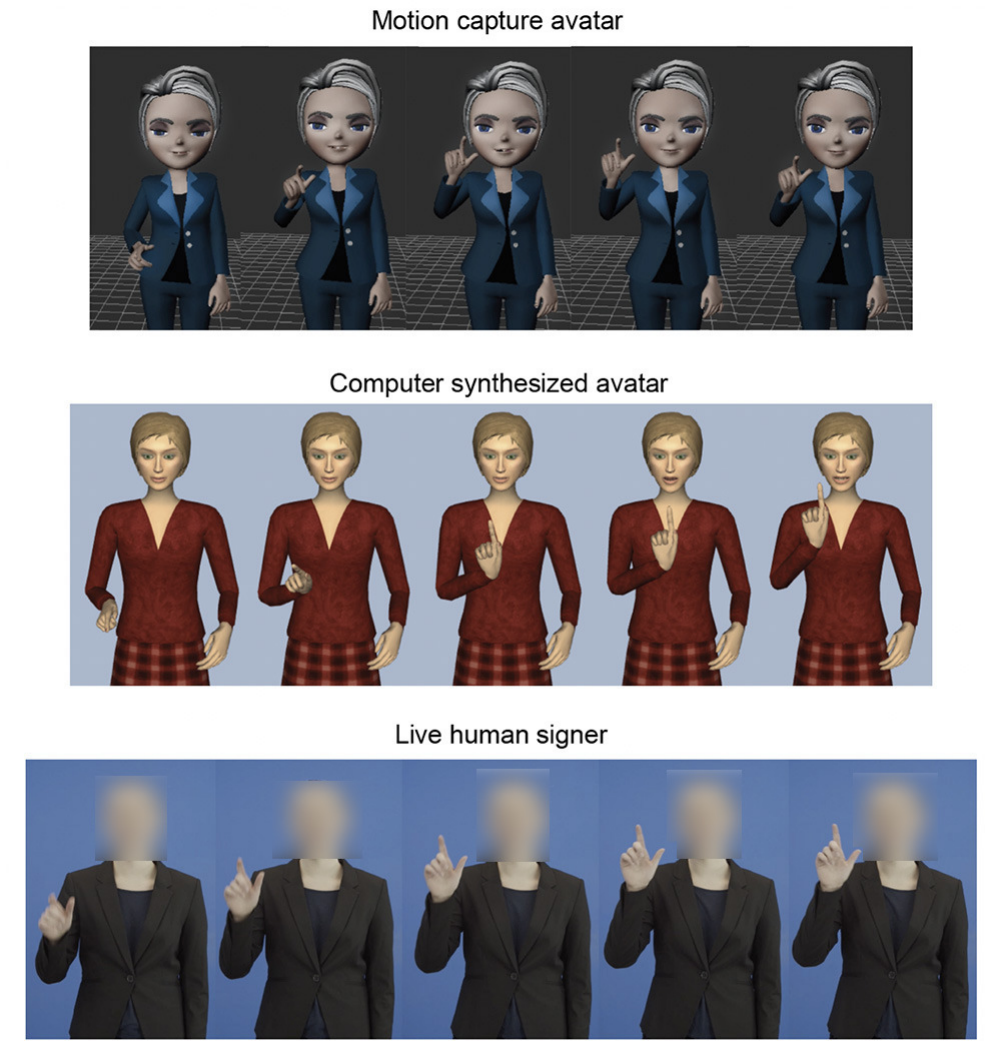
The three Signers producing the ASL sign LIBRARY. Top: avatar created using motion capture (Mocap avatar); middle: avatar created using SigML coding on the JASigning system (CS avatar); bottom: human signer. For this figure, one still frame was captured every three frames starting from approximate sign onset.

We created three types of video stimuli for the experiment (see Figure 1). Example videos of the three stimulus types are available at: https://doi.org/10.6084/m9.figshare.16877131.v2.

#### Human Signer

A female native ASL user was recorded against a blue background, producing each of the eight selected signs with natural sign production and facial expression. Each video clip started with 0.5 s of neutral body pose with arms at side, and then she signed one word. The clip ended at the end of the sign.

#### Computer-Synthesized Avatar

The same female native ASL user recorded for the human stimuli also coded the eight signs for the CS condition. She coded the movements of the computer-synthesized signing avatar (“Anna” character), created by JASigning, to produce the same eight signs as above. She used SigML-based coding input, which relies on the Hamburg Notation System (HamNoSys; Elliott et al., 2004). We recorded the screen to obtain video clips of each sign production, and each clip started with 0.5 s of neutral body pose and ended after the sign was complete.

#### Mocap Avatar

We created the motion-capture signing avatar according to methods described in more detail in Quandt et al. (2020). The motion capture data was recorded using a 16-camera Vicon motion capture system (Vicon Industries, Inc., Hauppauge, NY) with a custom-built Faceware Pro HD Mark 3.2 Headcam (Faceware, Austin, TX) facial expression camera. According to industry standards, one hundred twenty-three markers were placed on the signer's body, with labeling managed by Vicon Blade. Twenty-five markers were placed on each hand to ensure fidelity of hand movement information. The same female native signer who created the other stimuli produced eight signs during motion capture recording. We created eight video clips in which each clip started with ~0.5 s of a neutral body pose and ended after the sign was complete.

### Task

Participants took the survey online. Study data were collected and managed using REDCap electronic data capture tools hosted at Gallaudet University (Harris et al., 2009, 2019). REDCap (Research Electronic Data Capture) is a secure, web-based software platform designed to support data capture for research studies. The presentation of the signers and the specific sign rating items was randomized, and two different forms were created with different randomized orders. All items mentioned here are presented in detail in the Variables section. At the start of the survey, participants completed an informed consent form approved by the Gallaudet University Institutional Review Board. Next, participants answered several sets of questions. Before seeing any stimuli, participants answered questions about their general interest in signing avatars. Next, they saw a brief clip of each of the three Signers producing a short phrase (e.g., “Nice to meet you”) and rated their overall attitudes toward each Signer. In the next section, they saw the video clips of each Signer producing individual signs (described in section Video Stimuli). For each sign, they rated comprehension and naturalness. Following the individual sign ratings, each Signer was shown again, and participants gave ratings about their impressions of the Signer and rated them on several presumed characteristics. Finally, participants provided demographic information [birth date, sex, hearing status, preferred forms of communication, age of acquisition of ASL (Age of Acquisition), and self-reported fluency in ASL].

### Participants

Participants were recruited via online advertising, and we compensated them with a gift card in exchange for their time. Participants self-reported their hearing status, and we used those responses to group the participants. Eighty-three deaf, 34 hard-of-hearing, and 67 hearing ASL users were included in the sample. Table 1 shows participant demographics for each of the three groups. The table shows that Age of Acquisition and self-reported ASL fluency differed significantly between groups, with large effect sizes. On average, deaf participants reported the earliest Age of Acquisition and highest fluency with ASL. Hearing participants reported the latest Age of Acquisition, and *post-hoc* tests revealed the Hearing group had equivalent self-ratings of fluency as the Hard-of-Hearing group (*t* = 0.021, p_Tukey_ = 1.00).

**Table 1:** Participant demographics for three groups and statistical comparisons between groups.

	**Deaf**	**Hard-of-hearing**	**Hearing**	***F*_(2,181)_**	***p***	**eta2**
*N*	83	34	67	–	–	–
Age of ASL acquisition, years. M (SD)	5.74 (6.57)	10.66 (6.34)	17.21 (8.88)	43.72	<0.001	0.326
Self-reported ASL fluency; 1–5; M (SD)	4.56 (0.68)	3.76 (0.86)	3.76 (0.89)	23.23	<0.001	0.204
Age; M (SD)	31.25 (10.81)	29.44 (8.25)	28.96 (8.59)	1.23	0.294	0.01
				**χ^2^**		
Sex count; male, female, other	34, 47, 2	17, 15, 2	15, 50, 2	10.7	0.030	–

### Variables

A short introduction regarding signing avatars was presented: “*Virtual human characters can be made to communicate using signed languages. These “signing avatars” could potentially be used in many different areas of life. For example, a signing avatar may be able to translate spoken languages into sign language. In the future, you could see signing avatars when taking exams, watching the news, or when interacting with customer service*.” We then asked five questions regarding overall interest in signing avatars: *Signing avatars could be helpful…* (1)*…for understanding information on a website* [website]; (2)*…for communicating information in a public place (e.g., airport, train station)* [public place]; (3)*…as interpreters in a face-to-face meeting* [face-to-face]; (4) *I would enjoy seeing or interacting with signing avatars* [personal enjoyment]; (5) *Other people would enjoy seeing or interacting with signing avatars* [others' enjoyment]. These responses were given as 1-5 ratings, with Strongly Disagree as 1, Neutral as 3, and Strongly Agree as 5.

In line with our *a priori* predictions, we derived four new variables (Attitude, Impressions, Comprehension, and Naturalness) from the questionnaire by averaging responses to specific survey questions. The Attitude variable reflects averaged responses to the following, all of which were rated from 1 (strongly disagree) to 5 (strongly agree): *I would feel comfortable interacting with this signer*; *I would feel confident about receiving important information from this signer (e.g., news about COVID-19 or hurricanes)*; *I would trust the information I received from this signer*; and *I feel like I could share personal information with this signer (e.g., feelings, secrets, medical history)*. Cronbach's alpha showed high reliability between the individual items that made up the Attitude variable for all three Signers [Human: 0.76 (CI: 0.70–0.81); CS: 0.91 (CI: 0.88–0.93); Mocap: 0.90 (CI: 0.87–0.92)].

The Impressions variable reflects averaged responses to the following, using the same 1–5 rating scale as above: *This signer signs like a fluent deaf signer*; *This signer would be a good model of ASL for people who are learning to sign*; *This signer has appropriate use of facial expressions*; *This signer has appropriate use of body language*; *This signer's movements look clear*. Cronbach's alpha showed high reliability between the individual items that made up the Impressions variable for all three Signers [Human: 0.83 (CI: 0.79–0.87); CS: 0.93 (CI: 0.91–0.94); Mocap: 0.85 (CI: 0.81–0.88)].

In line with prior work (Kacorri et al., 2017), we calculated the Comprehension variable by averaging the responses to *This signing was easy for me to understand* for each of the eight individual signs produced by each signer. Cronbach's alpha showed high reliability between the individual items that made up the Comprehension variable for all three Signers [Human: 0.92 (CI: 0.90–0.94); CS: 0.94 (CI: 0.92–0.95); Mocap: 0.83 (CI: 0.79–0.86)]. The Naturalness variable was calculated by averaging the responses to *This signing looked natural* for each of the eight individual signs produced by each signer. Both questions used the same 1–5 rating scale described above. Cronbach's alpha showed high reliability between the individual items that made up the Naturalness variable for all three Signers [Human: 0.90 (CI: 0.88–0.92); CS: 0.98 (CI: 0.97–0.98); Mocap: 0.89 (CI: 0.87–0.91)].

As documented in our pre-registration, our analyses included within-group conditions based on the type of signer: Human signer, Mocap avatar, CS avatar. There were no between-group experimental condition assignments; however, we sorted the data into three groups based on self-reported demographics (Deaf, Hard-of-Hearing, Hearing). To conduct exploratory analyses, we also used the response to *What age were you when you first learned a sign language?* as the independent variable Age of Acquisition.

### Analyses

We analyzed data using JASP 0.14 analysis software (JASP Team, 2021). To examine the factors influencing overall interest in signing avatars, first we ran a two-way mixed ANOVA with one between-subjects factor (Group: Deaf, Hard-of-Hearing, Hearing) and one within-subjects factor (Scenario: Website, Public Place, Fact-to-Face). We then ran an exploratory Spearman's correlation between Age of Acquisition and overall attitudes toward signing avatars. We included the factors of Age of Acquisition, Website, Public Place, Face-to-face, Personal enjoyment, and Others' Enjoyment. Using a correlation allowed us to examine the continuous variable of Age of Acquisition to answer whether increasingly later Age of Acquisition is associated with any specific changes in interest or enjoyment of avatars.

To examine between-group differences and test our pre-registered predictions, we conducted a two-way mixed ANOVA with one between-subjects factor (Group: Deaf, Hard of Hearing, Hearing) and one within-subjects factor (Signer: Human, Mocap, and CS avatar) for each dependent variable based on our *a priori* predictions (Attitudes, Impressions, Comprehension, and Naturalness). Our data did not match the assumption of sphericity for these ANOVAs, so Greenhouse-Geisser-corrected *p*-values are shown for all analyses to account for this violation. To conduct an exploratory analysis on the relationship between Age of Acquisition and the ratings of the different signers, we conducted a Spearman's correlation, including the Age of Acquisition, and Comprehension, Naturalness, Attitude, and Impressions scores for all three signers. As above, using a correlation allowed us to examine the continuous variable of Age of Acquisition in more detail.

We also conducted two-way mixed ANOVAs using the between-subjects factor of self-reported Fluency in ASL (from 1 to 5 with 1 being not at all; 5 being extremely). We excluded any respondents who answered “1” from the dataset. We conducted these ANOVAs on the four planned primary dependent variables. Finally, we conducted an exploratory two-way mixed ANOVA on the ratings of how “creepy” each signer was because creepiness has a close link to the extensive literature on the uncanny valley effect (Mori et al., 2012; Kätsyri et al., 2015) and is commonly identified as one of the limiting factors of interacting with humanoid characters (Ho et al., 2008; Inkpen and Sedlins, 2011).

## Results

### Overall Ratings

**Figure 2:**
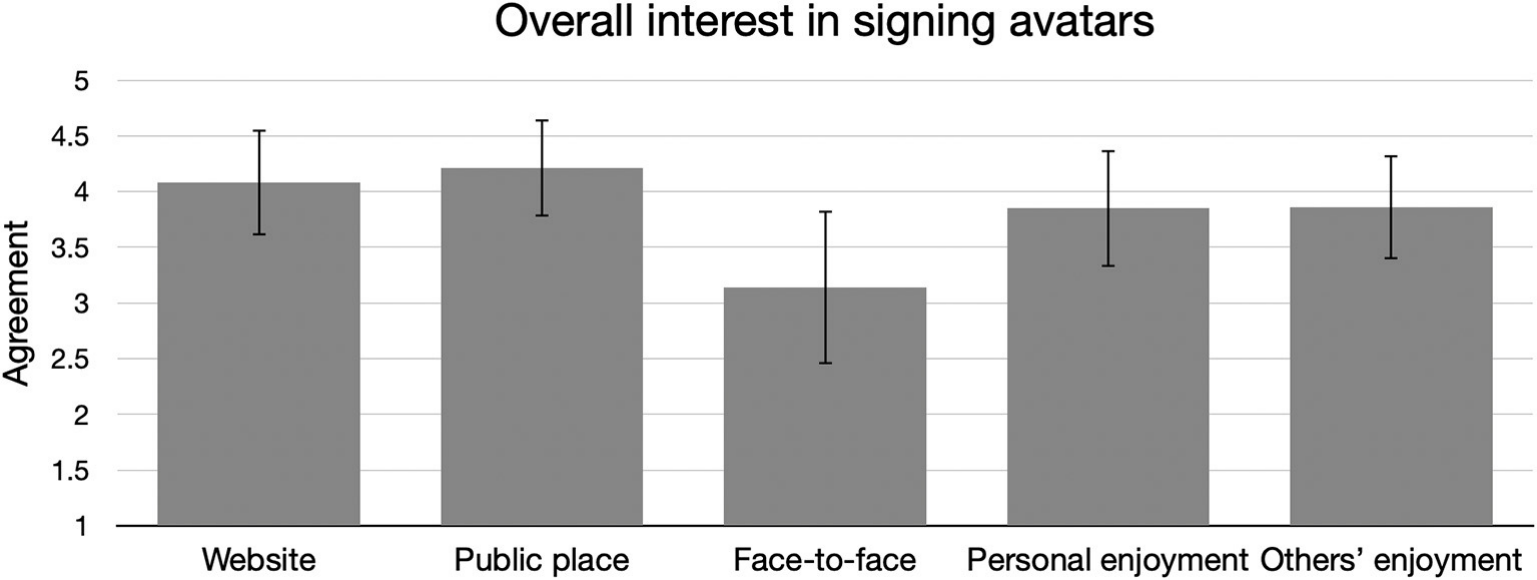
Responses across all participants, on agreement that signing avatars could be helpful for understanding information on a website, communicating information in a public place, or acting as an interpreter in a face-to-face meeting. The fourth and fifth columns show average responses across all respondents to whether the respondent would enjoy or expect others to enjoy seeing or interacting with signing avatars. 1 = strongly disagree - 5 = strongly agree, for all variables. Error bars show standard deviations.

Figure 2 shows the mean ratings across all participants for overall interest in signing avatars, both in three different scenarios, and the expectation of enjoyment for self and others. A two-way mixed ANOVA showed no significant differences between Groups on expressions of overall interest. There was a significant difference in interest regarding signing avatars in three different scenarios: Website, Public Place, and Face-to-Face [*F*_(1.67, 303.45)_ = 58.16, *p* < 0.001, eta2 = 0.12]. A Bonferroni-corrected *post-hoc* test revealed that Face-to-Face interest was significantly lower than either of the other two scenarios (*p*s < 0.001), while there was no difference between interest in Website or Public Place. The exploratory correlation between Age of Acquisition and overall interest ratings revealed a significant correlation between Age of Acquisition and ratings of personal enjoyment and others' enjoyment (see Table 2). In other words, people who acquired ASL later were more likely to express interest and enjoyment of avatars, whereas earlier learners reported less interest and enjoyment.

**Table 2:** Spearman's correlation between age of acquisition and overall acceptance of signing avatars.

**Spearman's correlations**							
**Variable**		**Age acq**.	**Website**	**Public place**	**Face-to-face**	**Personal enjoy**	**Others enjoy**
1. Age acq.	Spearman's rho	—					
2. Website	Spearman's rho	0.065	—				
3. Public place	Spearman's rho	0.030	0.408^***^	—			
4. Face-to-face	Spearman's rho	0.116	0.377^***^	0.178^*^	—		
5. Personal enjoy	Spearman's rho	0.190^**^	0.498^***^	0.374^***^	0.395^***^	—	
6. Others enjoy	Spearman's rho	0.170^*^	0.413^***^	0.383^***^	0.274^***^	0.566^***^	—

* *p < 0.05*,

** *p < 0.01*,

*** *p < 0.001*.

### Planned Analyses: Attitudes, Impressions, Comprehension, and Naturalness

**Figure 3:**
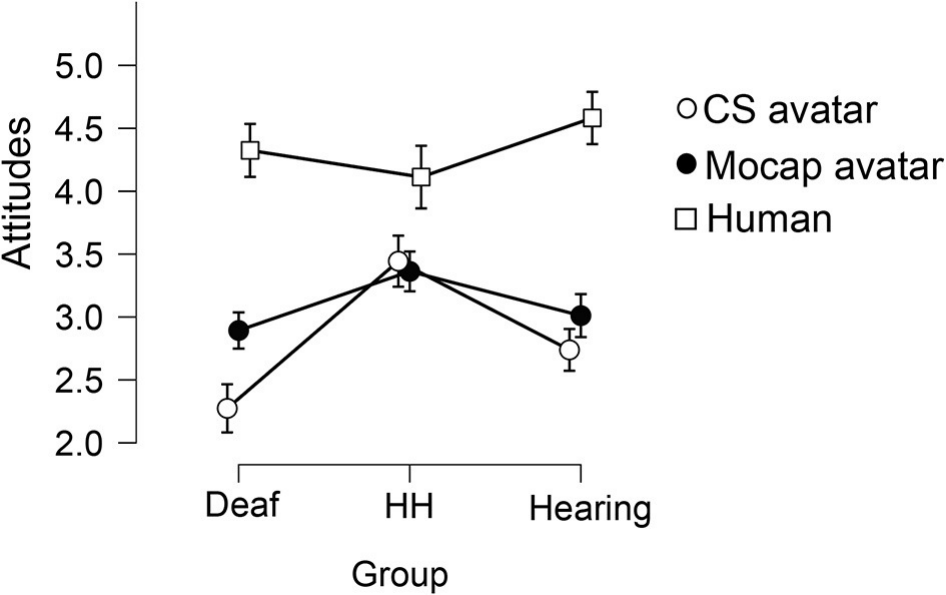
Average ratings of Attitudes toward the three Signers, between three Groups. The attitude rating reflects a respondent's willingness to accept important information from the signer and willingness to share information with the signer (for details, see Variables). Ratings could range from 1 (negative attitude) to 5 (positive attitude). Error bars show 95% confidence intervals.

A two-way mixed ANOVA was used to examine the effect of Signer type and Group on Attitudes, Impressions, Comprehension, and Naturalness. There was a small significant between-subjects effect of Group on Attitude ratings [*F*_(2, 181)_ = 7.46, *p* < 0.001, eta2 = 0.03], in which the Deaf group gave lower Attitude ratings overall (M = 3.17) than the Hard-of-Hearing group (M = 3.65) or Hearing group (M = 3.44). A Bonferroni-corrected *post-hoc* test revealed that while the Deaf group's Attitude ratings were significantly lower than the Hard-of-Hearing and Hearing groups (*p* = 0.001 and 0.03, respectively), the Hard-of-Hearing and Hearing groups did not differ. We observed a large significant within-subjects main effect of Signer [*F*_(1.72, 322.40)_ = 185.764, *p* < 0.001, eta2 = 0.32], in which the Human signer garnered the highest Attitude score (M = 4.40, SD = 0.617), followed by the Mocap avatar (M = 3.02, SD = 1.01), and the CS avatar garnered the least favorable Attitude score (M = 2.647, SD = 1.12). A Bonferroni-corrected *post-hoc* test revealed that Attitude ratings for all three Signers were statistically different from one another (all *p* < 0.005). We also observed a small significant interaction effect on Attitude ratings between Group and Signer [*F*_(3.56, 322.40)_ = 9.03, *p* < 0.001, eta2 = 0.03; see Figure 3]. A simple main effect follow-up test showed that the effect of Signer was significant for Attitude ratings of all three Groups (all *p* < 0.001). Simple main effects follow-up tests also showed that Attitude ratings varied significantly by Group for CS and Human Signers (*p* = 0.001 and 0.008, respectively), but not for the Mocap Signer (*p* = 0.09).

**Figure 4:**
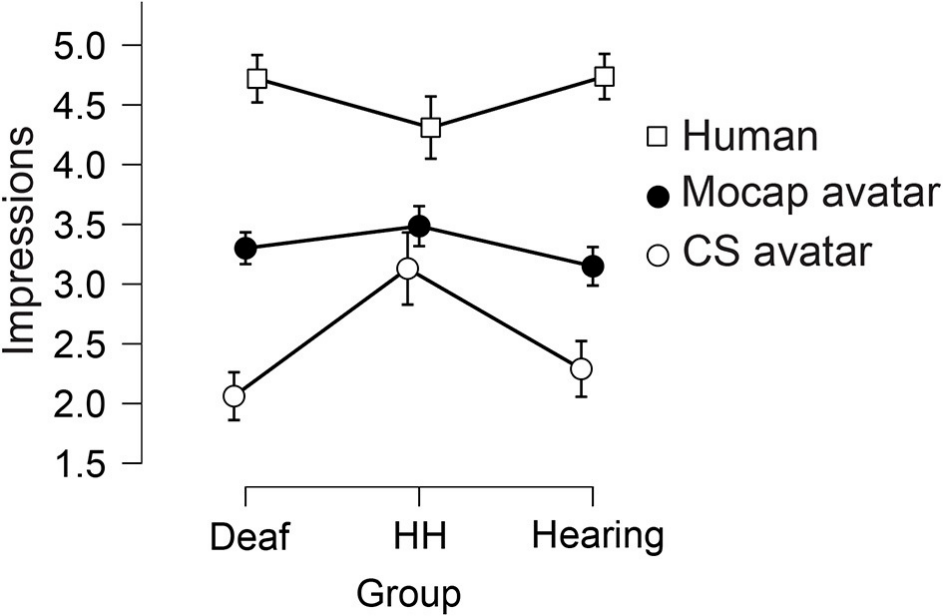
Average ratings of Impressions of the three Signers, between three Groups. The impression rating reflects a respondent's overall judgments of the signer's ASL fluency, movement quality, and expressiveness (for detail, see Variables). Ratings could range from 1 (negative impression) to 5 (positive impression). Error bars show 95% confidence intervals.

There was a small significant between-subjects effect of Group on Impression ratings [*F*_(2, 181)_ = 3.47, *p* = 0.03, eta2 = 0.008], in which the Hard-of-Hearing group gave higher Impression ratings (M = 3.65), while the Deaf group (M = 3.37) and the Hearing group (M = 3.39) gave lower ratings (see Figure 4). A Bonferroni-corrected *post-hoc* test revealed that the only significant difference between groups was that the Deaf group's Impression ratings were significantly lower than the Hard-of-Hearing group (*p* = 0.036), while other group comparisons were not significantly different. We observed a large significant within-subjects main effect of Signer [*F*_(1.67, 302.57)_ = 278.00, *p* < 0.001, eta2 = 0.46], in which the Human signer garnered the highest Impression score (M = 4.67, SD = 0.50), followed by the Mocap avatar (M = 3.28, SD = 0.05), and the CS avatar garnered the least favorable Impression score (M = 2.33, SD = 1.21). A Bonferroni-corrected *post-hoc* test revealed that Impression ratings for all three Signers were statistically different from one another (all *p* < 0.001). We also observed a small significant interaction effect on Impression ratings between Group and Signer [*F*_(3.43,302.57)_ = 10.05, *p* < 0.001, eta2 = 0.03; see Figure 4]. A simple main effect follow-up test showed that the effect of Signer was significant for Impression ratings of all three Groups (all *p* < 0.001). Simple main effects follow-up tests also showed that Impression ratings varied significantly by Group for CS and Human Signers (both *p* < 0.001), but not for the Mocap Signer (*p* = 0.163).

**Figure 5:**
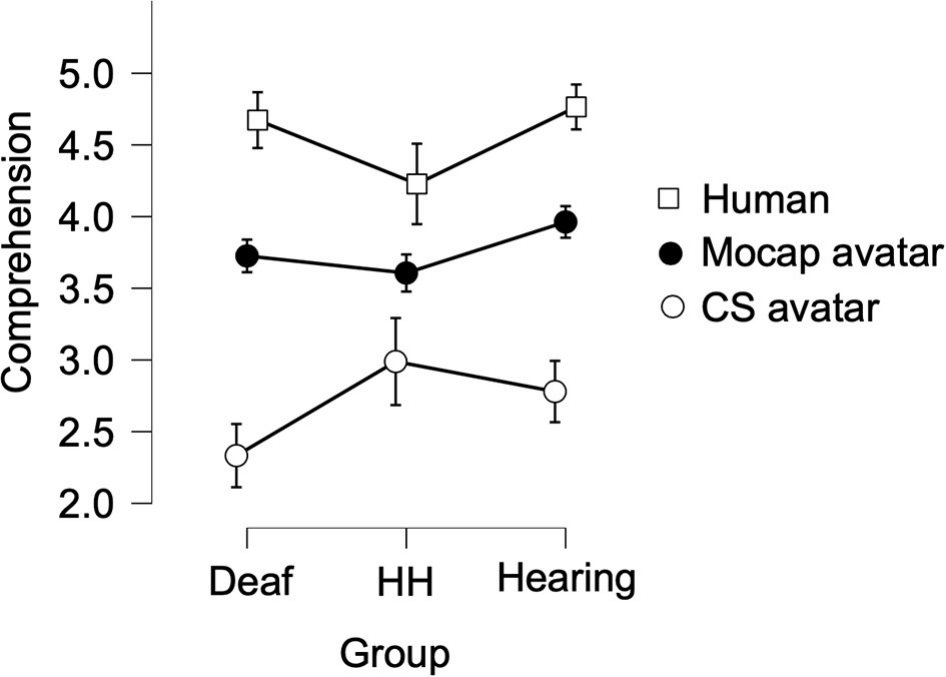
Average ratings of Comprehension of the three Signers between three Groups. The comprehension rating reflects the degree to which the respondent could understand each of the eight signs produced by each signer (for detail, see section Variables). Ratings could range from 1 (did not understand) to 5 (easy to understand). Error bars show 95% confidence intervals.

There was a small significant between-subjects effect of Group on Comprehension ratings [*F*_(2, 181)_ = 4.67, *p* = 0.01, eta2 = 0.012], in which the Hearing group (M = 3.86) gave higher Comprehension ratings, while the Deaf group (M = 3.57) and the Hard-of-Hearing (M = 3.61) group gave lower ratings. A Bonferroni-corrected *post-hoc* test revealed that the only significant difference between groups was that the Deaf group's Comprehension ratings were significantly lower than the Hearing group (*p* = 0.01), while other group comparisons were not significantly different. We observed a large significant within-subjects main effect of Signer [*F*_(1.39,250.96)_ = 239.76, *p* < 0.001, eta2 = 0.42], in which the Human signer garnered the highest Comprehension score (M = 4.62, SD = 0.56), followed by the Mocap avatar (M = 3.79, SD = 0.72), and the CS avatar garnered the lowest Comprehension score (M = 2.62, SD = 1.13). A Bonferroni-corrected *post-hoc* test also revealed that Comprehension ratings for all three Signers were statistically different from one another (all *p* < 0.001). We also observed a small significant interaction effect on Comprehension ratings between Group and Signer [*F*_(2.77, 250.96)_ = 6.72, *p* < 0.001, eta2 = 0.02; see Figure 5]. A simple main effect follow-up test showed that the effect of Signer was significant for Comprehension ratings of all three Groups (all *p* < 0.001). Simple main effects follow-up tests also showed that Comprehension ratings varied significantly by Group for all three Signers: CS (*p* = 0.006), Human (*p* < 0.001), and Mocap (*p* = 0.03).

**Figure 6:**
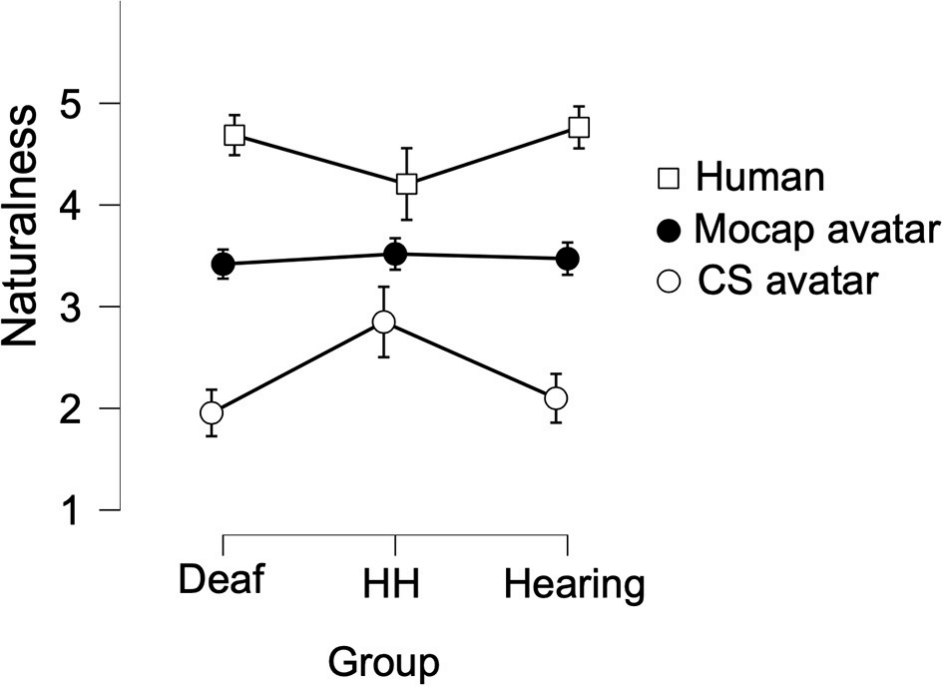
Average ratings of Naturalness of the three Signers, between three Groups. The naturalness rating reflects the degree to which each of the eight signs produced by each signer looked natural (for detail, see Variables). Ratings could range from 1 (unnatural) to 5 (natural). Error bars show 95% confidence intervals.

There was no main effect of Group on Naturalness ratings [*F*_(2,181)_ = 1.062, *p* = 0.348 eta2 = 0.003]. We observed a large significant within-subjects main effect of Signer [*F*_(1.59,287.42)_ = 272.55, *p* < 0.001, eta2 = 0.45], in which the Human signer garnered the highest Naturalness score (M = 4.62, SD = 0.56), followed by the Mocap avatar (M = 3.46, SD = 0.87), while the CS avatar received the lowest Naturalness ratings (M = 2.17, SD = 1.28). A Bonferroni-corrected *post-hoc* test revealed that Naturalness ratings for all three Signers were statistically different from one another (all *p* < 0.001). We also observed a small significant interaction effect on Naturalness ratings between Group and Signer [*F*_(3.18, 287.42)_ = 8.54, *p* < 0.001, eta2 = 0.03; see Figure 6]. A simple main effect follow-up test showed that the effect of Signer was significant for Naturalness ratings of all three Groups (all *p* < 0.001). Simple main effects follow up tests also showed that Naturalness ratings varied significantly by Group for the Human and CS Signers (both *p* < 0.002), whereas there was no difference based on Group for Naturalness ratings of the Mocap Signer (*p* > 0.8).

**Figure 7:**
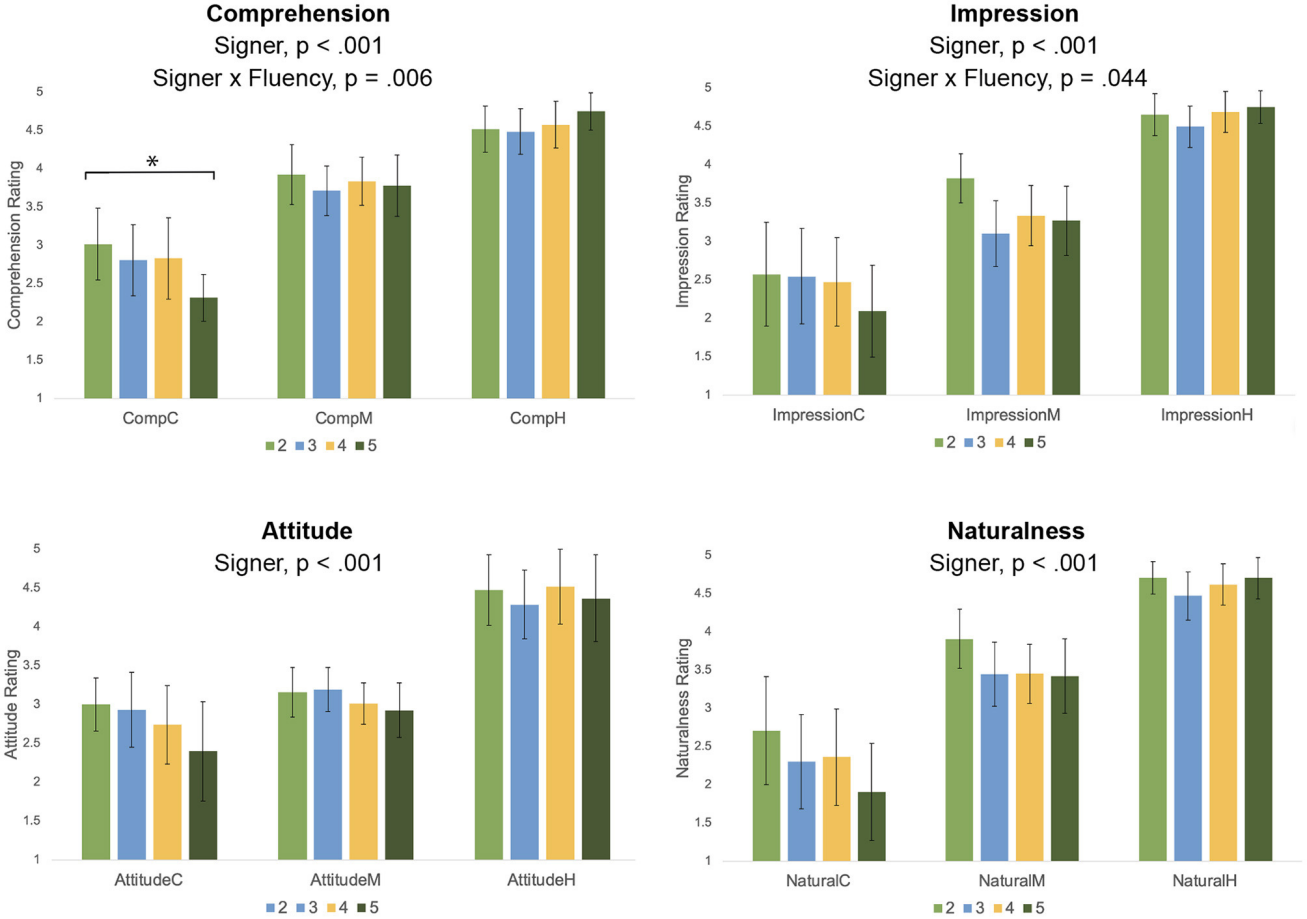
Fluency ratings (self-reported 1 = not at all to 5 = extremely; respondents who reported 1 were excluded) and ratings on Comprehension, Impression, Attitude, and Naturalness, for the three types of Signers (CS avatar, C; Mocap avatar, M; Human signer, H). Error bars show standard deviations. Asterix represents a significant effect of Fluency on Comprehension for the CS signer only.

We conducted two-way mixed ANOVAs to examine the relationship between Fluency and ratings on the four primary dependent measures (see Figure 7). For all four dependent measures (Comprehension, Naturalness, Impression, and Attitude), we observed a significant effect of Signer, in results that echo the consistent findings throughout this study. The Human garnered the highest ratings for all measures, the Mocap Signer was next highest, and the CS Signer was rated lowest. Of particular interest here, we asked to what extent these ratings varied based on the rater's self-reported ASL fluency. There was a small significant interaction effect on Comprehension ratings between Fluency and Signer [*F*_(4.142, 211.496)_ = 3.628, *p* = 0.006, eta2 = 0.02]. As seen in Figure 7, a Bonferroni-corrected simple main effects comparison showed that for only the CS Signer, Comprehension ratings decreased with increasing fluency [*F*_(3, 12.37)_ = 3.31, *p* = 0.021]. There was a small significant interaction effect on Impression ratings between Fluency and Signer [*F*_(4.879,292.715)_ = 2.33, *p* = 0.044, eta2 = 0.02; see Figure 7], but simple main effects comparisons did not yield any significant findings. For Attitude and Naturalness, there was no significant interaction effect.

### Exploratory Analysis: Creepiness

**Figure 8:**
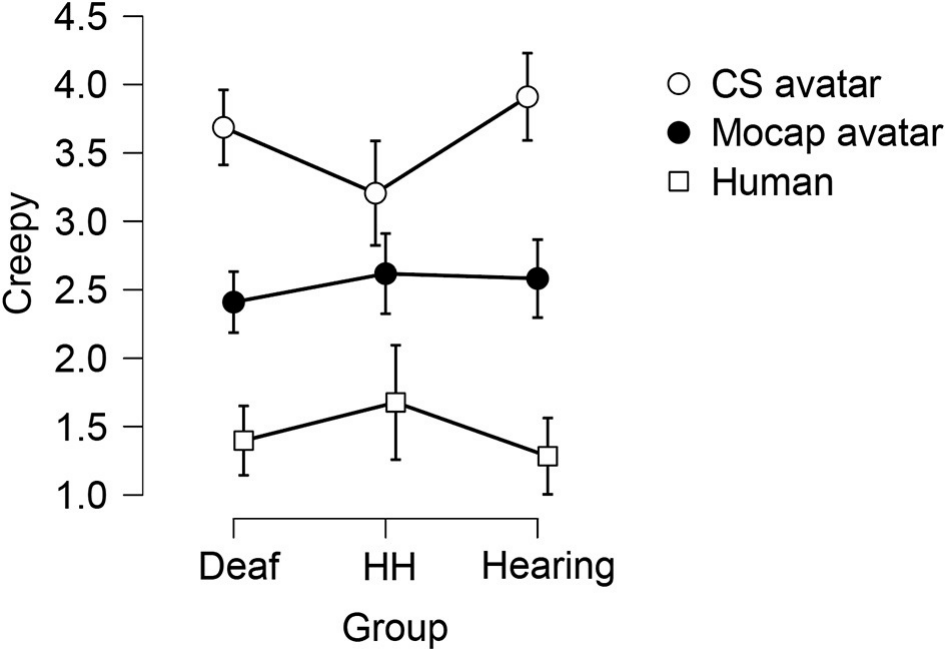
Average ratings of the Creepiness of the three Signers between three Groups. The creepiness rating reflects the degree to which each signer was judged to be “creepy” (for detail, see Variables). Ratings could range from 1 (strongly disagree) to 5 (strongly agree). Error bars show 95% confidence intervals.

There was no main effect of Group on Creepiness ratings [*F*_(2, 181)_ = 0.319, *p* = 0.727, eta2 < 0.001]. We observed a large significant within-subjects main effect of Signer [*F*_(1.92, 483.25)_ = 137.98, *p* < 0.001, eta2 = 0.31], in which the Human signer garnered the lowest Creepiness score (M = 1.46, SD = 0.98), followed by the Mocap Signer (M = 2.54, SD = 1.30), while the CS Signer received the highest Creepiness ratings (M = 3.61, SD = 1.35). A Bonferroni-corrected *post-hoc* test revealed that Creepiness ratings for all three Signers were statistically different from one another (all *p* < 0.001). We also observed a small significant interaction effect on Creepiness ratings between Group and Signer [*F*_(3.85,483.25)_ = 2.84, *p* < 0.026, eta2 = 0.01; see Figure 8]. A simple main effect follow-up test showed that the effect of Signer was significant for Creepiness ratings of all three Groups (all *p* < 0.001). Simple main effects follow up tests also showed that Creepiness ratings varied borderline-significantly by Group for the CS Signer (*p* = 0.045), whereas there was no difference based on Group for Naturalness ratings of the Human or Mocap Signers (both *p* > 0.1).

### Exploratory Analysis: Age of Acquisition

We ran an exploratory correlation between the four primary variables and users' Age of Acquisition to disentangle the age of ASL acquisition from Group membership and self-rated Fluency. The correlation showed that Age of Acquisition is significantly negatively correlated with Comprehension and Impression ratings of the Human signer; as the age of acquisition increases, the average Comprehension and Impression scores of the Human signer decreased (*p*-values of < 0.01; see Table 3). We also observed a significant positive correlation between Age of Acquisition and Attitude rating toward the Mocap avatar (*p* < 0.05). All four ratings of the CS signer (Comprehension, Naturalness, Attitude, and Impressions) were higher when the Age of Acquisition was later (*p*-values < 0.001). In other words, those who learned ASL later in life were more likely to give high ratings to the CS signer.

**Table 3:** Correlation between age of acquisition and comprehension, naturalness, impression, and attitude ratings for the three signers.

**Variable**	**Age acq**.	**CompH**	**NaturalH**	**AttitudeH**	**ImpH**	**CompM**	**NaturalM**	**AttitudeM**	**ImpM**	**CompC**	**NaturalC**	**AttitudeC**	**ImpC**
1. Age acq.	—												
2. CompH	−0.20^**^	—											
3. NatH	−0.13	0.80^***^	—										
4. AttH	−0.08	0.55^***^	0.57^***^	—									
5. ImpH	−0.23^**^	0.69^***^	0.71^***^	0.53^***^	—								
6. CompM	0.07	0.35^***^	0.32^***^	0.28^***^	0.26^***^	—							
7. NatM	0.09	0.09	0.14	0.15^*^	0.04	0.71^***^	—						
8. AttM	0.17^*^	−0.22^**^	−0.12	0.10	−0.16^*^	0.40^***^	0.62^***^	—					
9. ImpM	0.03	−0.10	−0.03	0.11	−0.04	0.44^***^	0.65^***^	0.65^***^	—				
10. CompC	0.39^***^	−0.42^***^	−0.37^***^	−0.15^*^	−0.40^***^	0.38^***^	0.38^***^	0.50^***^	0.34^***^	—			
11. NatC	0.35^***^	−0.53^***^	−0.43^***^	−0.19^*^	−0.45^***^	0.15^*^	0.39^***^	0.52^***^	0.39^***^	0.84^***^	—		
12. AttC	0.39^***^	−0.44^***^	−0.36^***^	−0.13	−0.40^***^	0.11	0.39^***^	0.62^***^	0.42^***^	0.65^***^	0.77^***^	—	
13. ImpC	0.33^***^	−0.51^***^	−0.46^***^	−0.20^**^	−0.48^***^	0.12	0.34^***^	0.51^***^	0.43^***^	0.77^***^	0.85^***^	0.71^***^	—

*Asterisks denote p-value thresholds*:

* *< 0.05*,

** *< 0.01*,

*** *< 0.001*.

## Discussion

The current study's purpose was to understand better what determines ASL users' responses toward signing avatars, considering both group-membership based on hearing status, and language background. We also wanted to see how different types of signing avatars would be judged by a heterogenous sample of signers. We designed an online rating study to gather responses from a large, diverse sample of ASL users, gathering information about their responses to different types of signers, as well as information about their own language use. Our primary hypothesis was that signing avatars created from motion capture recordings would elicit more positive attitudes and impressions, higher comprehension, and more natural signing ratings than computer-synthesized avatars (https://aspredicted.org/blind.php?x=uv6m9w). We also predicted that earlier Age of Acquisition and higher self-reported ASL fluency would result in less favorable views of signing avatars overall than people who learn ASL later in life or are less fluent.

The results presented here reflect a diverse array of responses. Participants included signers across a wide range of ASL fluency levels, and we gathered data from ASL users who were hearing, hard-of-hearing and deaf. The sample of participants was not limited to any one region or university, so it is unlikely that the cultural norms of a particular signing community are systematically over-represented. Taken together with the relatively large sample size, the wide variety of respondents means that our results may be more broadly generalizable than results from a study collected at a single location with a smaller sample size.

### Overall Responses to Signing Avatars

We investigated in which situations people were most interested in seeing or interacting with signing avatars. We asked participants to rate whether signing avatars would be helpful for understanding information on a website or for communicating information in a public place, and on average, respondents agreed that signing avatars would be helpful in those situations (see Figure 2). However, compared to other scenarios, a signing avatar acting as an interpreter in a face-to-face meeting was rated significantly worse, which we did not find surprising due to face-to-face interactions' fluid and personal nature. These findings reinforce the notion that signing avatars hold the most promise—and stand to be most accepted by the community—in circumstances where rote, impersonal information is being shared (WFD and WASLI, 2018; Bragg et al., 2019). There was no significant difference between groups on these ratings. There were no significant relationships between Age of Acquisition and responses to the situation-related questions, suggesting that perceptions of helpfulness in different scenarios do not differ based on language background and hearing status. The correlation did reveal a higher likelihood of enjoying interactions with signing avatars as the Age of Acquisition increases (see Table 2), suggesting that later ASL learners are generally more open to signing avatars.

### What Determines Responses to Signing Avatars?

#### Intrinsic Factors

In this study, we compared three types of signers: a human native ASL user (Human), an avatar created using computer-synthesized motion (CS Signer), and an avatar created using motion capture recording (Mocap Signer). We included these three signers to compare two examples of different types of avatars against a high-level control of a native human signer, whom we expected to garner relatively high ratings on all dependent measures. Across the four primary variables (Attitudes, Impressions, Comprehension, and Naturalness), respondents gave the human signer the highest ratings, the Mocap avatar the next highest, and the CS avatars the lowest ratings. For these four variables, the within-group differences between Signers were quite large. These findings confirm our pre-registered predictions that signing avatars created from motion capture recordings would elicit more positive attitudes and impressions, higher comprehension, and more natural signing ratings than computer-synthesized avatars. On all factors, people rated the Human significantly higher than the Mocap avatar, suggesting that while they viewed the Mocap avatar more positively than the CS avatar, fluid motion alone is not sufficient to garner higher responses; visual appearance matters as well. Even when an avatar moves like a human, people respond less positively if it does not look human. Taken together, the differences between the avatars suggest that this Mocap avatar presented a significant advantage over the CS avatar, while at the same time, neither avatar is viewed as positively as a real human signer.

Attitudes, Impressions, Comprehension, and Naturalness each garnered similar patterns of responses. The Attitudes variable included questions about whether people would feel comfortable sharing or receiving information from the signer (see section Variables). Respondents had very positive attitudes toward the human signer (M = 4.40 out of 5), whereas they reported significantly less interest in exchanging information with either kind of avatar. The Impressions variable measured visual judgments of how the signer moved, looked, and signed (see section Variables). Respondents had very positive impressions of the Human's movements and signing (M = 4.67 out of 5), and the Mocap avatar garnered lower Impression responses, which were still significantly better than Impressions of the CS avatar. The Comprehension and Naturalness measures showed how well each Signer was understood and whether their sign production was natural or not. Both the Comprehension and Naturalness variables followed the same pattern again, with large significant differences between signers. Lastly, we conducted the exploratory analysis on Creepiness and found that as predicted, the CS avatar was judged as the creepiest, while the Mocap avatar was less creepy, and as expected, the Human signer garnered very low creepiness ratings. Taken together, we see resounding support for the predicted differences between avatars. We show that compared to the CS avatar, ASL users overall had more positive attitudes and impressions of the Mocap Signer, who they also found to be more comprehensible and natural.

#### Extrinsic Factors

We were particularly interested in how a person's own language background would influence their ratings of the avatars. Overall, we saw significant effects of Group on Attitude, Impression, and Comprehension, but no effect of group on Naturalness or Creepiness. The Deaf group gave significantly lower Attitude ratings than the other two groups. In contrast, for Impression ratings, the difference was driven by the Hard-of-Hearing group giving significantly higher ratings than the Deaf group. For Comprehension, the difference was driven by the Hearing group giving significantly higher ratings than the Deaf group.

Overall, we see that while hearing status is related to people's responses to signing avatars, the exact nature of that relationship varies depending on what type of characteristic they are judging. For example, the Hearing group's higher Comprehension ratings may reflect their lower ASL fluency, resulting in more forgiveness for unclear or unnatural movements because they lack the necessary knowledge to discriminate between different productions of a sign. In the current work, we had about half as many Hard-of-Hearing respondents (*N* = 34) as we did in the Deaf and Hearing groups. This smaller group size, as well as the wide range of experiences which may lead people to identify as hard-of-hearing (Luey et al., 1995; Israelite et al., 2002), means that the responses from this group are hard to interpret in comparison to either the Deaf or Hearing groups.

#### Intrinsic and Extrinsic Factor Interactions

To understand signers' feelings toward signing avatars more thoroughly, we also looked at interactions between intrinsic and extrinsic factors. Specifically, we asked how people's language background and hearing status are related to the ratings of the different types of signers. These analyses were motivated by the idea that more fluent signers, especially deaf signers, may be more sensitive to signing avatars' movement dynamics and facial expressions than people who are newer or less fluent users of ASL, as has been suggested by prior literature (Kacorri et al., 2015, 2017). We observed small but significant Group x Signer interactions for all four primary factors and the exploratory factor of Creepiness. Though many specific differences were driving the interactions (see Figures 3–7; section Planned Analyses: Attitudes, Impressions, Comprehension, and Naturalness), the Hard-of-Hearing group showed different responses for many variables. For Attitudes, Impressions, Naturalness, and Creepiness, the Hard-of-Hearing group responded more positively to the CS Avatar than the other two groups and more negatively to the Human signer. We observed that the Hard-of-hearing group displayed a different profile of responses than the Deaf group, but the interpretation of these differences is limited due to the diversity of the sample and the smaller size Hard-of-Hearing group as noted above. Comprehension ratings showed that the Deaf group judged the CS Avatar less comprehensible than the other two groups.

In addition to analyzing Group differences, we also conducted analyses using self-reported Fluency as an independent variable and correlations with the Age of Acquisition variable. While Group and Fluency are certainly related, as seen in the significantly higher Fluency ratings for the Deaf group on average (Table 1), analyzing by Fluency gives us a clearer picture of how proficiency in ASL, rather than hearing status alone, is related to the perceptions of signing avatars. This distinction is important because, within the Groups, fluency varies widely—for instance, the Deaf group includes many people whose first language is ASL and who are highly fluent, yet also includes later, less proficient Deaf learners of ASL. From the Group x Fluency analyses, we observed that higher Fluency was related to lower Comprehension ratings for the CS Signer (see Figure 7). For Impressions, we saw the same pattern in which people with higher ASL fluency had more negative Impressions toward the CS Signer.

The correlation between Age of Acquisition and the four primary variables mirrored this finding. For all four variables, people with an earlier Age of Acquisition rated the CS avatar more negatively (see Table 3). These findings are essential because they show that more fluent and earlier signers are particularly sensitive to the odd movements of the CS avatar. In contrast, Fluency was not related to Comprehension or Impression ratings for the Mocap avatar (confirmed by *post-ho*c testing, *p*-values of 0.80 and 0.15, respectively), suggesting that those ratings of the Mocap avatar do not differ based on the viewer's own fluency. We suggest that this may be because the Mocap avatar's movements come from a recording of a human native signer. While she does not look realistically human, and there may be features of her appearance that people find odd, her sign production reflects the authentic signing movements of a native Deaf signer. This finding adds to prior work suggesting that people are highly sensitive to the motion characteristics of humanoid agents (White et al., 2007; Saby et al., 2011; Thompson et al., 2011). This work also echoes past findings in the field, which demonstrated that ASL users who attended Deaf Residential schools (where ASL is the primary language) were more harsh judges of computer-synthesized ASL animations (Kacorri et al., 2017).

The different experiences of Deaf, Hard-of-Hearing, and Hearing people who use ASL and the differences based on their fluency in ASL are critical to consider when developing signing avatars. Users' language experiences appear related to their responses to signing avatars, and critically, certain types of signing avatars may be more prone to negative responses from particular groups of people. When developing signing avatars, one key aspect of design considerations must be who the intended audience or user will be (Bragg et al., 2019). Our data show that deaf or fluent signers are likely to have more negative views of signing avatars, especially when animated with computer-synthesized motion. At the same time, developers must consider the vast diversity of the deaf experience when designing signing avatars for use in any particular situation. For instance, many deaf and hard-of-hearing people do not grow up in an environment where signed language is used (Mitchell and Karchmer, 2004) and do not receive signed language exposure during early developmental years (Kushalnagar et al., 2011). Some of them may later choose to learn sign language, and people in this group represent an important demographic for signing avatars. As later ASL learners, as shown by our data, those people may have a more accepting view of signing avatars.

These complex relationships between the user's background and the avatar they see are essential to understand if avatars are used in high-stakes educational, medical, or face-to-face settings. The work we present here echoes work from other domains of human-computer interaction, in which the preference for more authentic human experience drives the acceptance of virtual human assistants (Chérif and Lemoine, 2019; Fernandes and Oliviera, 2021).

## Limitations and Future Directions

This study replicates specific findings from the literature, particularly the more negative responses from more fluent signers (Kacorri et al., 2015) and the complex multidimensional factors which affect users' views of signing avatars (Kipp et al., 2011). We also go beyond prior work to better portray the complex relationship between people's own language background and the way they approach and react to different types of signing avatars. However, our results only represent the specific virtual human avatars used here, so extrapolation beyond these instances should be taken with caution. Dozens of different signing avatars have been developed over the years, and it is beyond the scope of the study to draw conclusions about those not included in this study. Another limitation of the current work is that most ratings were given in response to individual words, so more work is needed to understand how signers respond to transitions between signs and grammatical aspects of avatar signing.

Signing avatar technology changes quickly. Since the stimulus creation in 2019-early 2020, advances in motion capture, facial expression recording, and computer synthesis of signed language have all continued to accelerate rapidly, so newer versions of signing avatars may not encounter the same responses like the ones included here. Achieving greater success with signing avatars may come from cross-disciplinary collaboration, such as ensuring that technical development occurs with meaningful input from signed language linguistics (Bragg et al., 2019). Researchers in signed language linguistics have had a long ongoing discussion on best representing signed languages in notation systems (Miller, 2001; Hochgesang, 2014). Continued collaboration between sign linguists and avatar developers may allow for improved representation of sign language by virtual humans. Our current findings, as well as several ethical considerations recently discussed in the field (Bragg et al., 2019; De Mulder, 2021; Krausneker and Schügerl, 2021; Quandt and Malzkuhn, in review^1^), suggest that deaf signers should serve as the models for signing avatars to ensure quality and cultural relevance. Finally, it would be helpful to carry out a similar study with a cross-design in which avatars varied by appearance and movement type. Such a study would allow for comparison between responses to two avatars who look the same but whose motions are different and compare responses to two avatars who look different but whose motions are the same.

## Conclusion

In the work presented here, we found that movement quality and appearance significantly impact users' ratings of signing avatars and that signed language users with earlier Age of Acquisition (and more fluency) are the most sensitive to the movement quality issues seen in avatars based on computer-synthesized motions. Given that this sensitivity was not evident for the Mocap avatar, we suggest that developers of signing avatars must retain the fluid movements integral to signed languages. Since people who learn ASL earlier are more likely to rely on it as a primary language, interference of computer-generated movements into signed content is unlikely to be met with acceptance by the users for whom signing avatars may be the most useful.

## Data Availability Statement

The raw data supporting the conclusions of this article will be made available by the authors, without undue reservation.

## Ethics Statement

The studies involving human participants were reviewed and approved by Gallaudet University Institutional Review Board. The patients/participants provided their written informed consent to participate in this study.

## Author Contributions

LQ conceptualized and supervised the study, conducted formal analysis and visualization, acquired funding, drafted and wrote the work. AW contributed to the conceptualization, methodology, investigation, and review/editing of the work. MS contributed to conceptualization, methodology, data curation, and review/editing of the work. KW contributed to the methodology, investigation, and review/editing of the work. RF contributed to investigation and review/editing of the work. All authors contributed to the article and approved the submitted version.

## Funding

We recognize support from National Science Foundation Grants #1839379 and #2118742 and Gallaudet University's VL2 Center.

## Conflict of Interest

The authors declare that the research was conducted in the absence of any commercial or financial relationships that could be construed as a potential conflict of interest.

## Publisher's Note

All claims expressed in this article are solely those of the authors and do not necessarily represent those of their affiliated organizations, or those of the publisher, the editors and the reviewers. Any product that may be evaluated in this article, or claim that may be made by its manufacturer, is not guaranteed or endorsed by the publisher.

## Footnotes

^1^Quandt, L. C., and Malzkuhn, M. (in review). The Way Forward for Signing Avatars: Seven Principled Recommendations.
